# Identification and Biocontrol of Pathogenic Fungi Causing Root Rot of *Polygonatum cyrtonema* Hua

**DOI:** 10.3390/jof12070483

**Published:** 2026-07-01

**Authors:** Zi-Xin Wang, Yan-Xi Chen, Xin-Pei Ye, Zhong-Bao Jiang, Wen-Qing Xia, Yu-Hang Zhou, Shu-Qi Chen, Qin Zhu, Lu-E Shi

**Affiliations:** 1 Department of Biotechnology, College of Life and Environmental Sciences, Hangzhou Normal University, Hangzhou 311121, China; 2 College of Life and Environmental Sciences, Hangzhou Normal University, Hangzhou 311121, China; 3 Zhejiang Provincial Modern Biology and Medicine Industry College, Hangzhou Normal University, Hangzhou 311121, China

**Keywords:** *Polygonatum cyrtonema* Hua, isolation, identification, *Fusarium*, antagonism

## Abstract

*Polygonatum cyrtonema* (*P. cyrtonema*) Hua is an important economic crop with both edible and medicinal value. However, frequent root rot severely restricts its industrial development, resulting in sharp yield reduction and quality deterioration. To clarify the primary pathogenic fungi causing root rot of *P. cyrtonema* Hua, 58 fungal strains from naturally diseased *P. cyrtonema* Hua plants in different habitats were isolated in this study. By combining morphological observation and molecular identification based on 18S rDNA and ITS rDNA sequences, the species of 22 pathogenic fungi were identified, among which 10 strains belonged to the genus *Fusarium*, accounting for 45.45% of the identified isolates. The pathogenicity of 21 pathogenic fungi was verified according to Koch’s postulates, with findings indicating that *Fusarium* species exhibited significant pathogenic potential. Meanwhile, six previously identified endophytic *Paenibacillus* strains isolated from *P. cyrtonema* Hua were employed to perform dual culture assays and antifungal evaluations of their fermentation supernatants against representative strains including *F. concentricum* F2, *Neopestalotiopsis* sp. F3 and *F. oxysporum* F8. The results indicated that the antagonistic activity exhibited by the six strains exceeded 50%, with the inhibition rates of their fermentation supernatants against strains F2, F3 and F8 surpassing 73%. This study confirmed that *Fusarium* is the dominant pathogenic fungal group causing root rot of *P. cyrtonema* Hua. Furthermore, highly effective antagonistic endophytes were preliminarily identified, offering candidate strains and a theoretical foundation for the green management of root rot in *P. cyrtonema* Hua.

## 1. Introduction

*Polygonatum cyrtonema* Hua belongs to the genus *Polygonatum* of the *Liliaceae* family, and is a traditional food–medicine homologous herb in China. Its rhizomes are rich in various active components such as polysaccharides, steroidal saponins, flavonoids, and alkaloids [1,2], and have various medicinal values such as immune regulation, anti-tumor, anti-fatigue, anti-depression, and lowering blood sugar [1,3,4]. In recent years, with the widespread recognition of the concept of “dietary therapy,” the market demand for edible and medicinal homologous plants such as *P*. *cyrtonema* Hua has continuously increased. However, *P*. *cyrtonema* Hua is highly susceptible to diseases during planting, transportation, and storage, among which root rot is one of the most important [5]. Since the rhizome is the key part of *P*. *cyrtonema* Hua to exert its main values [1], the occurrence of root rot will lead to a significant severe yield losses and quality degradation, which has become one of the bottlenecks restricting the sustainable development of *P*. *cyrtonema* Hua resources.

Existing studies have shown that *Fusarium* is one of the dominant pathogenic groups causing root rot of *Polygonatum* plants [6,7]. Xu et al. [8] isolated *F*. *avenaceum* from root rot tissues of wild and greenhouse-cultivated *P*. *cyrtonema* Hua in Sichuan Province, China. An et al. [9] isolated *F*. *oxysporum* from rotten tubers of *P*. *cyrtonema* Hua in Guizhou Province, China. Wei et al. [10] first identified *F*. *commune* as a novel pathogen causing rhizome rot of *P*. *cyrtonema* Hua. In addition, Gong et al. [11] found that *T*. *virens* could cause leaf chlorosis of *P*. *cyrtonema* Hua leaves accompanied by rhizome rot. However, current studies mostly focus on a single production area or specific pathogen, lacking systematic investigations of pathogens causing root rot of *P*. *cyrtonema* Hua in different habitats. Moreover, to control pathogenic fungi causing rhizome rot of *P*. *cyrtonema* Hua, chemical control is generally employed. At present, chemical pesticides such as carbendazim, chlorothalonil, mancozeb, and prochloraz are widely used in the control of root rot of medicinal plants [12]. Chemical control is also the main prevention and control method for root rot of *P*. *cyrtonema* Hua. Although it can suppress disease symptoms in the short term, long-term use is likely to lead to pathogen resistance, soil microecological imbalance, and excessive pesticide residues in medicinal materials [5]. By contrast, biological control has become a research hotspot in recent years due to its environmental friendliness and strong sustainability. Many studies have shown that plant endophytes can inhibit pathogens by secreting antimicrobial substances and competing for ecological niches [13,14], but the screening of high-efficiency antagonistic endophytes against root rot of *P*. *cyrtonema* Hua is still in its infancy.

Our research group previously isolated 359 endophytes from *P. cyrtonema* Hua. Among these endophytes, 56 strains exhibiting antibacterial activity against *Escherichia coli*, *Staphylococcus aureus*, and *Bacillus subtilis* were obtained, among which 42 strains showed superior antimicrobial effects. These strains provided high-quality endophyte resources for subsequent targeted antagonistic screening against the pathogenic fungi of *P. cyrtonema* Hua. Root rot disease significantly impedes the large-scale cultivation of *P. cyrtonema* Hua, while existing investigations on its pathogenic microbes lack systematic multi-habitat field surveys. Given the above limitations, *P. cyrtonema* Hua samples were collected from various cultivation habitats in Zhejiang Province, China, and employed as experimental materials in the present study. This approach facilitated the systematic isolation and identification of root rot pathogens, aiming to further elucidate the pathogen composition of *P. cyrtonema* Hua root rot in the local area. Meanwhile, in contrast to previous studies that mostly used exogenous endophytes for antifungal evaluations, six antimicrobial endophytic strains originally isolated from healthy *P. cyrtonema* Hua during our prior screening were selected to perform antagonism assays against the identified pathogens, including *Paenibacillus* sp. 3-11 (PZ501727), 3-29 (PZ501728), 3-62 (PZ501729), 3-65 (PZ501730), T26-2 (PZ501731) and T45-2 (PZ501732). These investigations would enhance the adaptability and targeted biocontrol efficacy, providing a scientific foundation for the management of *P. cyrtonema* Hua root rot, and promoted its green development.

## 2. Materials and Methods

### 2.1. Sample Collection and Processing

**Figure 1:**
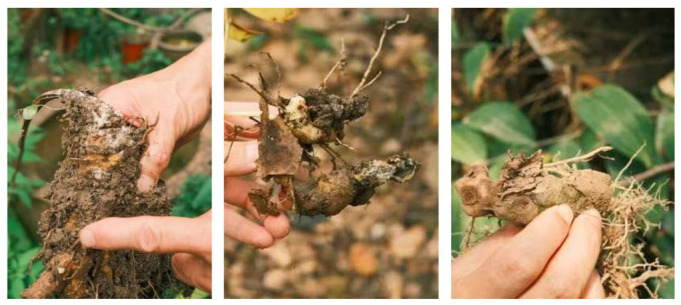
Decay of *P. cyrtonema* Hua.

In July 2024, naturally diseased tissues of *P*. *cyrtonema* Hua were collected from diverse growth environments within its production areas in Zhejiang Province, China, including pine forest cultivation, greenhouse cultivation, potted cultivation, and rock crevice cultivation. Plants exhibiting typical symptoms of root rot disease (Figure 1) were selected, such as rhizome browning and rot, shedding of epidermis, wilting and yellowing of leaves. Samples were immediately placed in sterile preservation bags, which were labeled with the habitat type, diseased location, and collection date, and then transported to the laboratory for refrigeration at 4 °C. Subsequent processing was completed within 24 h.

Samples from different parts of naturally infected *P. cyrtonema* Hua were selected. After washing off surface soil, the samples were disinfected with 75% ethanol for 5 min in a clean bench, then soaked in 2.0% sodium hypochlorite for 5 min. Finally, they were rinsed three times with sterile water, and dried on sterile filter paper to remove surface moisture, thus completing surface disinfection [15].

### 2.2. Isolation and Purification of Pathogens

The pathogens were isolated from various diseased tissues [16]. A 5 mm × 5 mm tissue fragment was excised at the lesion–healthy tissue interface and inoculated onto potato dextrose agar (PDA) medium, consisting of 200 g of potatoes, 20.0 g of glucose, 15.0 g of agar, and 1000 mL of pure water. Four tissue fragments were placed per plate, with 3 replicates per sample. The final rinse solution was spread onto PDA medium as a control. Plates were incubated at 28 °C for 3–5 d, with daily observation of colony growth. After colonies grown on the medium, fresh hyphae from the edges of colonies exhibiting different morphologies were picked and transferred to fresh PDA plates for repeated purification until pure strains were acquired.

### 2.3. Identification of Pathogenic Fungi

#### 2.3.1. Morphological Observation

Purified pathogenic fungi were inoculated onto PDA medium and incubated at 28 °C in the dark for 6 d. The morphological properties such as the color and texture of colonies on the plates were then observed. Hyphae of the pathogenic fungi were scraped off and their hyphal morphology and spore characteristics were examined under a microscope.

#### 2.3.2. Molecular Identification

Mycelia cultured on PDA medium were collected, thoroughly mixed with sterile water, and utilized as the templates. The primers used for amplification were ITS1-F: 5′-TCCGTAGGTGAACCTGCGG-3′; ITS4-R: 5′-TCCTCCGCTTATTGATATGC-3′; NS1-F: 5′-GTAGTCATATGCTTGTCTC-3′; NS6-R: 5′-GCATCACAGACCTGTTATTGCCTC-3′ [17]. The amplification system consisted of the following: 1.0 μL template, 12.5 μL 2 × SanTaq PCR Mix Premix, 1.0 μL F primers (10 μM), 1.0 μL R primers (10 μM), and 9.5 μL ddH_2_O, with a total reaction volume of 25 μL. The amplification program was as follows: initial denaturation at 94 °C for 5 min, followed by 35 cycles of denaturation at 94 °C for 30 s, annealing at 53 °C for 30 s, and extension at 72 °C for 60 s, with a final extension at 72 °C for 10 min.

A 1.2% agarose gel was prepared with the addition of 1 × TAE buffer. Subsequently, agarose gel electrophoresis was performed at 150 V and 100 mA for 20 min to detect the presence of target bands [18]. Following this, the PCR products were sent to Sangon Biotech Co., Ltd. (Shanghai, China) for sequencing. The resulting sequences were analyzed using NCBI WebBLAST searches against the NCBI homologous sequence database to identify the species of pathogenic fungi infecting *P. cyrtonema* Hua [19,20].

### 2.4. Pathogenicity Test

The pathogenicity of tested fungal isolates was assessed following Koch’s postulates [21]. Fresh *P. cyrtonema* Hua rhizomes and leaves were rinsed three times with sterile saline solution and blotted dry with sterile filter paper. Surface disinfection was performed sequentially with 75% ethanol for 2 min and 2.0% NaClO solution for 5 min, then rinsed with sterile water and air-dried naturally. Holes were punched on the surface of the disinfected and air-dried healthy *P. cyrtonema* Hua rhizomes using a sterile punch. Purified pathogenic mycelial plugs with a diameter of 8.0 mm were inoculated onto the wounded sites [22]. Holes were made in the middle of the leaves, and then the wounds were inoculated with hyphae of the pathogenic fungi. Sterile saline solution was used as the negative control, with three replicates for each treatment. Samples were wrapped with plastic wrap and placed in sterile fresh-keeping boxes [23,24], stored at room temperature. Tissue changes and disease occurrence were observed every 2 d for 8 consecutive days. Based on the decay status, the fungi were confirmed as the primary rot-causing pathogens. Subsequently, fungi were re-isolated from rotten tissues to verify their consistency with the original isolates. Morphological identification was employed to confirm the strains in this study.

### 2.5. Preliminary Screening of Endophytes with Antagonistic Activity

The tested endophytes consisted of six strains of *P. cyrtonema* Hua endophytes with significant antimicrobial activity, which were previously isolated by our group (strain numbers: 3-11, 3-29, 3-62, 3-65, T26-2, T45-2; stored at −80 °C). All endophytes were streaked and activated on PDA medium, and then incubated at 28 °C for 24 h. According to the method of Cao et al. [25], the two-point plate confrontation method was employed. A pathogenic fungal mycelial plug of 8.0 mm diameter was inoculated at the center of a PDA plate, while the endophytes were spot-inoculated at 2.0 cm away from the center. The centers of the two inoculation sites were collinear. Sterile water was used as the control, with three replicates for each treatment. All plates were incubated at 28 °C for 6 d. Following incubation, the unrestricted hyphal radius (R_0_) of the pathogenic fungi and the antagonism-inhibited radius (R_1_) were measured along the connecting line of the two inoculation points. The inhibition rate (IR) was calculated using the formula: IR (%) = [(R_0_ − R_1_)/R_0_] × 100%.

### 2.6. Activity Test of Endophytes Fermentation Supernatant

According to the method described by Zhang et al. [26], a single colony of the activated endophytes from Section 2.5 was picked and inoculated onto potato dextrose broth (PDB) medium, consisting of 200 g of potatoes, 20.0 g of glucose, and 1000 mL of pure water. The fermentation was carried out at 28 °C at 160 r/min for 3 d. The culture was centrifuged at 10,000 r/min for 10 min to collect the supernatant, which was then filtered through a 0.22 μm membrane filter to obtain sterile fermentation supernatant. The sterilized PDA medium was cooled to approximately 55 °C, after which the sterile fermentation supernatant was added to PDA medium to achieve a final concentration of 10% (*v*/*v*). PDA medium supplemented with an equal volume of sterile water was used as the control. After thorough mixing, the medium was poured into sterile Petri dishes to prepare PDA plates containing different sterile fermentation supernatants. A pathogenic mycelial plug of an 8.0 mm diameter was inoculated at the center of each plate, with three replicates for each treatment. All plates were incubated at 28 °C for 6 d. The colony radius of pathogenic fungi in the control group was defined as R_0_, and that in the fermentation supernatant treatment group was defined as R_1_. The inhibition rate was calculated using the formula in Section 2.5.

### 2.7. Data Analysis

All data were analyzed using Excel 2024 to calculate the mean values and standard deviations. Statistical analysis was performed using SPSS 27.0. Before the analysis, Levene’s test for homogeneity of variances was conducted. One-way analysis of variance (ANOVA) combined with Tukey’s HSD multiple comparison test was applied, with a significance level of *α* = 0.05. Image assembly was carried out using Adobe Illustrator 2025.

## 3. Results

### 3.1. Isolation and Identification of Pathogenic Fungi

#### 3.1.1. Overview of Isolated Strains

A total of 58 fungal strains were isolated from the collected diseased *P*. *cyrtonema* Hua tissues, with strain sources covering six categories (Table 1). Among them, the largest number of strains was isolated from greenhouse-cultivated *P*. *cyrtonema* Hua (28 strains, accounting for 48.28%), followed by processed and stored samples (15 strains, 25.86%), potted cultivated (6 strains, 10.34%), wild-simulated cultivated (4 strains, 6.90%), rock crevice cultivated (3 strains, 5.17%), and pine forest cultivated (2 strains, 3.45%). According to the statistical analysis of disease sites, the largest number of strains was isolated from tuber tissues (36 strains, accounting for 62.07%).

#### 3.1.2. Morphological Observation

**Figure 2:**
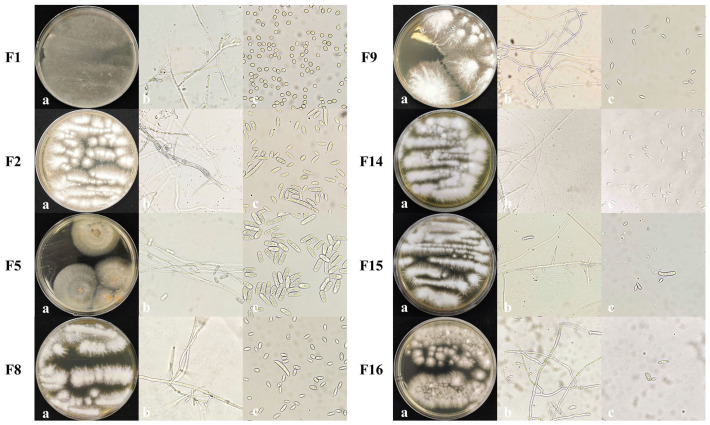

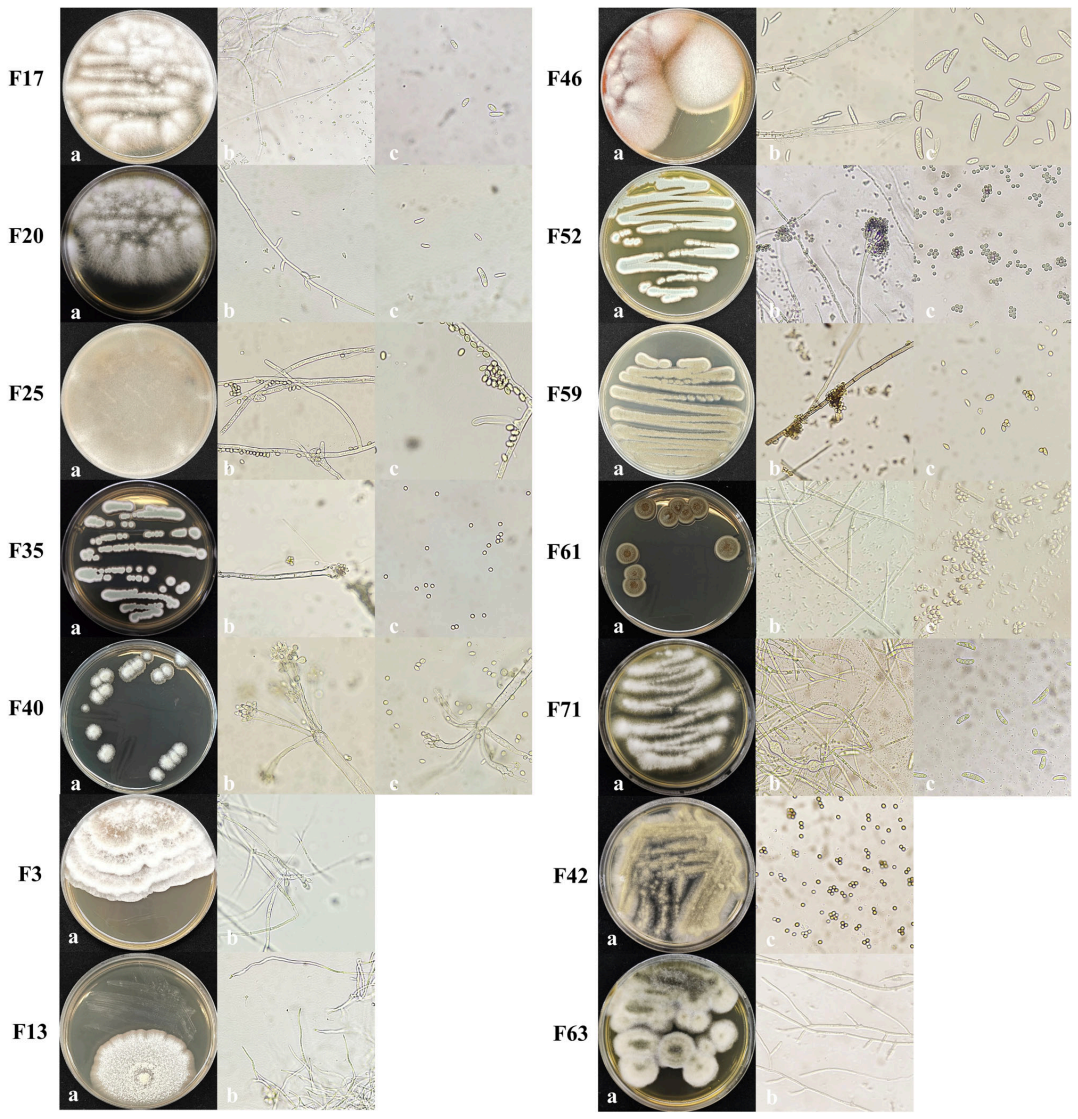
Morphology of colony (**a**), mycelium (**b**) and conidia (**c**) of the fungal pathogens of *P. cyrtonema* Hua.

The 58 isolated pathogenic fungal strains of *P. cyrtonema* Hua exhibited significant differences in colony morphology, and 22 representative isolates were selected for morphological observation (Figure 2). Most colonies were white (F2, F3, F8, F9, F13, F14, F15, F20, F40), off-white (F1, F5, F17, F25) or milky white (F16, F71), whereas a small proportion of isolates produced brown (F42, F46, F59, F61, F63) and grey-green colonies (F35, F52). The textures included flocculence, villosity, and a powdery texture. Most colonies exhibited radial growth with a loose and expansive morphology. Several isolates formed circular, patch-shaped colonies (F35, F40, F61), while a few demonstrated special concentric ring characteristics (F3).

Among the 22 isolates, F1, F35, F40, F42 and F52 produced small globose conidia. Isolates F5 and F25 exhibited elliptical conidia with variations in size. The conidia of isolates F59 and F61 were subglobose with slightly pointed terminals. Isolates F3, F13 and F63 did not produce detectable conidia, while the remaining isolates produced falcate conidia. The hyphae of most pathogenic fungi were septate with obvious branches, and a few were non-septate. Specialized structures such as broom-like branches and sporangiophores were observed in isolates F1, F35, F40 and F52.

#### 3.1.3. Molecular Identification for Fungal Pathogens

**Figure 3:**
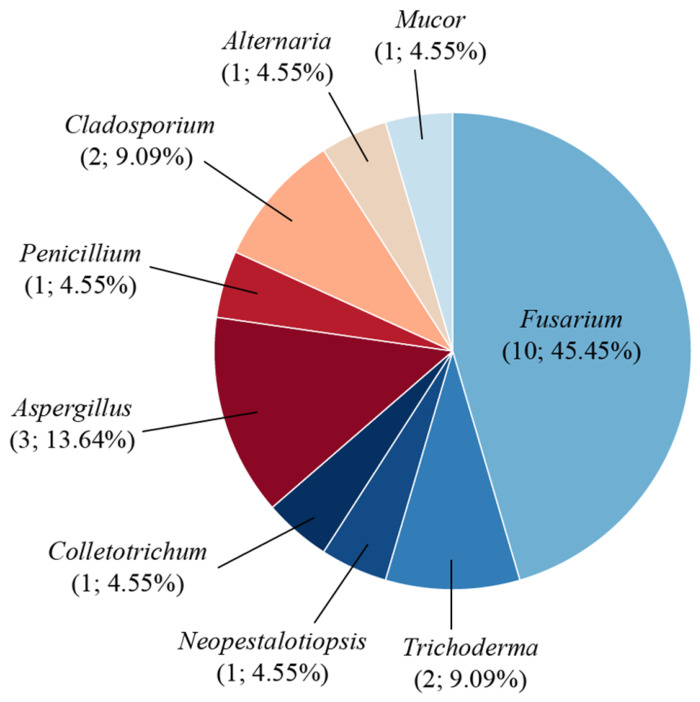
Genus distribution of pathogenic fungi from diseased *P. cyrtonema* Hua.

Based on morphological observations, 22 representative isolates with distinct colony morphology, hyphal characteristics, and conidial characteristics were selected for PCR amplification and sequencing of the 18S rDNA and ITS regions. The obtained sequences were submitted to the NCBI database, and their corresponding accession numbers were listed in Table 2. Sequence alignments of the sequencing results were performed using the BLAST. High sequence identity with reference strains enabled species-level classification of 21 isolates. Strain F9 shared only 86.56% identity with its top hit *Mucor odoratus* and was classified as *Mucor* sp. in this study due to unconfirmed taxonomic status. All 22 pathogenic isolates were classified into 9 genera. Among them, *Fusarium* was the dominant genus, accounting for 45.45%, followed by *Aspergillus* (Figure 3).

### 3.2. Pathogenicity Verification

**Figure 4:**
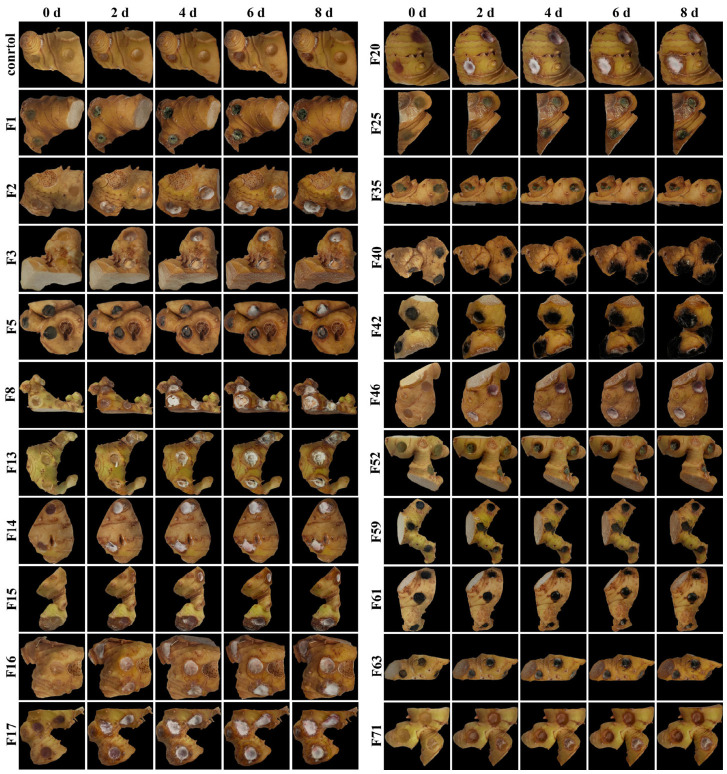
Pathogenicity test of fungal pathogens on healthy *P. cyrtonema* Hua rhizomes.

Based on the molecular identification results and the representativeness of their habitats, 21 characterized isolates were subjected to pathogenicity reinoculation assays, while strain F9 was excluded due to its unresolved taxonomic status. The results indicated that the control group only showed natural senescence, with water loss at the rhizome incision and slight browning, but no disease symptoms. All experimental groups showed typical disease symptoms, which worsened over time (Figure 4). Among the 21 tested strains, most *Fusarium* strains (F2, F8, F13, F14, F15, F16, F17, F20) exhibited strong pathogenicity. After inoculation, white hyphae grew on the rhizome, and the tissue turned brown, softened, and rotted. Over time, the lesions changed from light brown to dark brown and expanded continuously, accompanied by a distinct rotten odor. Several strains of the genus *Aspergillus* (F40, F42) also exhibited high pathogenicity. The inoculated rhizomes developed severe lesions, with tissues extensively colonized by mycelia and severely softened.

**Figure 5:**
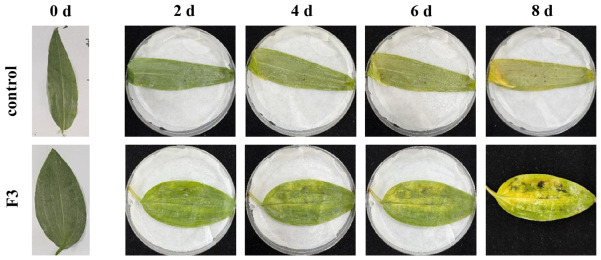
Pathogenicity test of fungal pathogens on healthy *P. cyrtonema* Hua leaves.

A pathogenicity test of strain F3 on *P*. *cyrtonema* Hua leaves was also conducted. The leaves of the control group gradually turned chlorotic over time, accompanied by slight yellowing. After inoculation with strain F3, the inoculation site gradually turned black, eventually forming black spots, and the extent of yellowing around the inoculation site was greater than that in other areas (Figure 5). Microorganisms were re-isolated from the healthy–diseased junction tissues of *P. cyrtonema* Hua after reinoculation. The strains after isolation and purification were consistent with the inoculated strains, which conformed to Koch’s postulates.

### 3.3. Screening of Endophytes with Antagonistic Activity

#### 3.3.1. Determination of Antagonistic Activity via the Dual Culture Method

**Figure 6:**
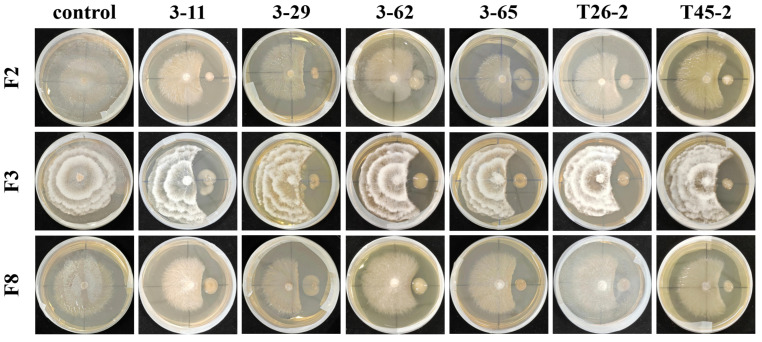
Inhibitory effects of six endophytic strains of *P. cyrtonema* Hua on three kinds of pathogenic fungi.

The six tested endophytic bacteria of *P. cyrtonema* Hua exhibited different degrees of antagonistic activity against strains F2, F3 and F8 (Figure 6). Among them, the inhibitory effect of the strains on strain F3 was the strongest, with the inhibition rate ranging from 71.49 to 79.33%, and there were no significant difference in the inhibitory effect among strains (*p* > 0.05). The inhibition rate on strain F2 ranged from 54.91 to 66.35%, and there was a significant difference in the inhibitory effect among strains (*p* < 0.05), indicating that there was significant variation in the antagonistic potential of the tested endophytes against this pathogen. The inhibition rate on strain F8 ranged from 56.41 to 67.71%, and there were significant differences among strains (*p* < 0.05). Strain 3-29 had the strongest broad inhibitory effect on strains F2, F3, and F8, with an average inhibition rate of 70.27%. Strains 3-11 and 3-65 were ranked in second place, with both achieving an average inhibition rate of 69% against the three pathogenic fungi. Among them, strain 3-65 had particularly prominent inhibitory effects on strains F2 and F3, with inhibition rates of 66.35% and 79.33%, respectively (Table 3).

#### 3.3.2. Antagonistic Activity of Fermentation Supernatant of Endophytic Strains

**Figure 7:**
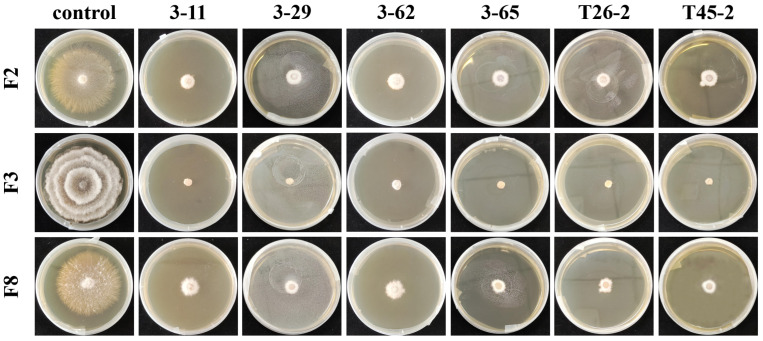
Inhibitory effects of fermentation supernatant of six endophytes of *P. cyrtonema* Hua on three kinds of pathogenic fungi.

The results showed that the fermentation supernatants of the six tested endophytic strains from *P. cyrtonema* Hua exhibited significant antagonistic activity against strains F2, F3 and F8 (Figure 7). Among them, the inhibitory effect of the endophytic strains against strain F3 was the strongest, with inhibition rates ranging from 87.42 to 90.0%. Except for endophytic strain 3-62, the inhibition rates of other strains all reached 90.0%, and there were extremely significant differences among strains (*p* < 0.001). The inhibition rates against strain F2 ranged from 76.11 to 80.20%, and there were extremely significant differences among strains (*p* < 0.001). The inhibition rates against strain F8 ranged from 73.59 to 79.52%, and there were extremely significant differences among strains (*p* < 0.001). Endophytic strain T26-2 had the strongest broad inhibitory effect on strains F2, F3, and F8, with the highest inhibition rate. The antagonistic effects of endophytic strains 3-11, 3-29, 3-65, and T45-2 were similar. The antagonistic effect of endophytic strain 3-62 was relatively poor, and its inhibition rates against strains F3 and F8 were lower than those of other strains, but still remained above 70% (Table 4). The six endophytic strains of *P. cyrtonema* Hua show potential as candidate strains for the biological control of *P. cyrtonema* Hua root rot.

## 4. Discussion

### 4.1. Dominance of Pathogenic Fungal Community

*Fusarium* is the dominant pathogenic fungal community causing root rot disease in *P*. *cyrtonema* Hua and other *Polygonatum* species, among which *F*. *oxysporum* and *F*. *solani* have been specifically identified as the primary rot-causing pathogens of this disease [6,7]. Among the 22 pathogenic strains isolated and identified in this study, 10 strains belonged to *Fusarium*. *F*. *oxysporum* F8, F16, F46 and *F*. *solani* F13, F71 were successfully isolated, which exactly aligned with these two groups of primary pathogens. Moreover, pathogenicity tests further confirmed that *F*. *oxysporum* F8 and F16 could cause typical symptoms in *P*. *cyrtonema* Hua rhizomes, including browning, softening, and rapid lesion expansion accompanied by a putrid odor, demonstrating significant rot-causing capability. Lu et al. [27] analyzed the rhizosphere microbial community and found that besides *Fusarium*, the dominant fungal species in the rhizosphere soil of *P*. *cyrtonema* Hua with root rot also included *Mucor* and *Trichoderma*. In this study, we isolated strain F9 (closely related to *M. odoratus* with 86.56% sequence identity, yet its taxonomic status remained unconfirmed), *T*. *virens* F1 and *T. spirale* F25, which exactly corresponded to these two groups. Although *T*. *virens* and *T. spirale* are commonly regarded as biocontrol fungus, Dautt-Castro et al. [28] confirmed that they could transform into pathogenic fungi in the absence of specific factors, suggesting that they might act as potential pathogens of *P*. *cyrtonema* Hua. Meanwhile, the present study is one of the first to report the presence of *F*. *concentricum* F2, a highly pathogenic root rot fungus from *P*. *cyrtonema* Hua. This fungus related to root rot of *P*. *kingianum* has only been reported previously by Yang et al. [29]. Our findings further expanded the pathogenic host range of *F*. *concentricum* in *Polygonatum* species. Strain F3 was identified as *Neopestalotiopsis* sp., which could cause strawberry root rot [30] and plant leaf spot diseases [31,32], and was also the pathogen responsible for grey leaf spot disease in *P*. *cyrtonema* Hua [33]. *Neopestalotiopsis* sp. F3 was isolated from yellow leaves of *P*. *cyrtonema* Hua. It could cause both leaf disease and slight depression and rot in rhizome tissues, representing the first confirmation that it could act directly as a primary pathogen to cause root rot in *P*. *cyrtonema* Hua, rather than merely a secondary pathogen associated with foliar diseases.

The distribution of pathogenic fungi exhibited significant variation across different habitats. Strains isolated from *P*. *cyrtonema* Hua under greenhouse cultivation accounted for 48.28%, which was significantly higher than that from wild habitats such as under-forest and rock crevice environments. This phenomenon may be related to differences in secondary metabolite contents and soil microenvironments of *P*. *cyrtonema* Hua across different habitats. Studies have indicated that forest-cultivated *P*. *cyrtonema* Hua contains higher amounts of polysaccharides and mineral elements than greenhouse and container-cultivated *P*. *cyrtonema* Hua [34]. Polysaccharides can enhance plant disease resistance and inhibit the growth of pathogens [35]. Al-Hazmi et al. [36] reported that polysaccharide treatment alleviated bacterial diseases and *Fusarium*-induced crown wilt in tomato plants. Furthermore, *P*. *cyrtonema* Hua requires cultivation in well-drained soil with adequate water retention. Greenhouse environments are characterized by high humidity and poor ventilation, while container cultivation is associated with insufficient drainage, thus resulting in a higher isolation rate of pathogenic fungi. This habitat-specific distribution pattern (characterized by pathogen enrichment in artificial cultivation and sparsity in wild habitats) is a key driver of frequent root rot outbreaks in the artificial cultivation of *P. cyrtonema* Hua, reflecting a specific association between pathogens and cultivation practices.

### 4.2. Preliminary Screening of Antagonistic Endophytes

The six endophytic bacteria from *P. cyrtonema* Hua, including *Paenibacillus* spp. 3-11, 3-29, 3-62, 3-65, T26-2 and T45-2, demonstrated excellent biocontrol capacities. In comparison with strains reported in recent studies, these endophytic strains displayed good antifungal activity and significant potential for practical application. Zheng et al. [37] reported that in the confrontation tests, the inhibition rates of strains QZ2, QZ8 and WK1 against *F. oxysporum*, the primary pathogenic fungus of *P. cyrtonema* Hua, were 59.6%, 31.97% and 10.60%, respectively, and these rates were lower than those of most strains in this study. Li et al. [5] reported that the endophyte *B. velezensis* Y24 isolated from *Polygonatum* exhibited an inhibition rate of approximately 70.63% against the pathogen *F. oxysporum* in confrontation tests. The most potent strain 3-29 in our study showed slightly lower activity compared with Y24. All six biocontrol strains demonstrated inhibition rates exceeding 73% in the fermentation supernatant antagonism assay. In addition, the antifungal spectra of most currently available biocontrol agents for the prevention and control of *P. cyrtonema* Hua root rot did not cover pathogenic fungi *F. concentricum* and *Neopestalotiopsis* sp. The endophytic strains identified in this study demonstrated strong inhibitory effects against these two groups of pathogenic fungi, exhibiting broader application potential. In summary, these strains are expected to become candidate strains for replacing chemical fungicides and achieving green control of *P. cyrtonema* Hua root rot.

## 5. Conclusions

This study systematically identified the primary pathogens and highly efficient antagonistic endophytic resources associated with *P*. *cyrtonema* Hua root rot while uncovering the distribution characteristics of pathogens and the antifungal potential of endophytes across different habitats. In total, 58 fungal strains were isolated from diseased *P*. *cyrtonema* Hua across different habitats. Koch’s postulates were confirmed for 21 of these strains, all of which were found to be pathogenic to the rhizomes of *P*. *cyrtonema* Hua. The fermentation supernatants of six endophytes exhibited inhibition rates exceeding 73% against core pathogenic fungi, demonstrating potent antifungal activity. This study provides key target strains and a critical theoretical foundation for developing green control strategies for *P*. *cyrtonema* Hua root rot.

## Figures and Tables

**Table 1:** Sources of pathogenic fungi of *P. cyrtonema* Hua.

Source Category	Symptom Description	Strain Number	Quantity/Strains
Cultivated under pine forest	*P*. *cyrtonema* Hua tubers with red spots	F1	1
	*P*. *cyrtonema* Hua with leaf disease	F29	1
Cultivated under greenhouse	*P*. *cyrtonema* Hua tubers with red spots	F4, F25, F49, F50, F7, F11, F14, F15, F16, F17, F19, F35, F37, F62, F71	15
	*P*. *cyrtonema* Hua fibrous roots with red spots	F6, F20, F27, F38, F66	5
	*P*. *cyrtonema* Hua tubers with blight	F5, F10, F42, F55	4
	Wilted *P*. *cyrtonema* Hua tubers	F30, F32	2
	*P*. *cyrtonema* Hua rhizome and roots	F45	1
	*P*. *cyrtonema* Hua tubers	F47	1
Cultivated under rock crevice	Yellow leaves of *P. cyrtonema* Hua	F3, F43	2
	White mold-like tissue on *P*. *cyrtonema* Hua fibrous roots	F12	1
Cultivated under potted conditions	White mold-like tissue in the middle of *P*. *cyrtonema* Hua tubers	F13	1
	White mold-like tissue on the left part of *P*. *cyrtonema* Hua tubers	F28, F46, F57	3
	White mold-like tissue on the upper-middle part of *P. cyrtonema* Hua tubers	F36	1
	White mold-like tissue on the upper part of *P. cyrtonema* Hua tubers	F41	1
Cultivated under simulated wild conditions	Fruits of *P. cyrtonema* Hua	F2, F8, F31, F33	4
Processed and stored environments	Rotten root tissues of *P. cyrtonema* Hua	F9, F24, F26, F39	4
	Rotten *P. cyrtonema* Hua tissue	F23, F51, F72	3
	External white mold-like tissues of rotten *P*. *cyrtonema* Hua	F56	1
	Moldy *P*. *cyrtonema* Hua tubers without nine steaming and nine processing	F40, F48, F52, F53, F59, F61, F63	7
Total			58

**Table 2:** BLAST results of the fungal pathogens of *P. cyrtonema* Hua in the GenBank.

Fungal Strain	GenBank Accession Number	The Closely Related Ex-Type Strain	Query Cover (%)	E Value	Percent Identity (%)	Accession Length
F1	PZ245564	*Trichoderma virens* strain Z2 (OQ629197.1)	100	0.0	99.83	588
F2	PZ326464	*Fusarium concentricum* strain NCJD4 (PP825349.1)	100	0.0	100.00	561
F3	PZ326442	*Neopestalotiopsis* sp. isolate PPNLRSA_PATUASR (OR553981.1)	100	0.0	99.82	551
F5	PZ260813	*Colletotrichum gloeosporioides* isolate F4P2Fe (ON026110.1)	99	0.0	99.63	1360
F8	PZ326443	*Fusarium oxysporum* strain D23B2 (PQ836409.1)	100	0.0	99.64	562
F9	PZ501690	*Mucor odoratus* strain CBS:120.71 (MH860028.1)	98	0.0	86.56	634
F13	PZ326441	*Fusarium solani* strain QT113(5) (OQ818151.1)	100	0.0	99.47	569
F14	PZ245561	*Fusarium circinatum* CBS 405.97 (NR_120263.1)	97	0.0	98.12	560
F15	PZ245565	*Fusarium pseudoanthophilum* CBS 414.97 (NR_163682.1)	98	0.0	98.16	557
F16	PZ260815	*Fusarium oxysporum* isolate F4P4Lg (ON026118.1)	99	0.0	99.78	1357
F17	PZ245562	*Fusarium circinatum* CBS 405.97 (NR_120263.1)	97	0.0	98.13	560
F20	PZ245566	*Fusarium circinatum* CBS 405.97 (NR_120263.1)	99	0.0	98.66	560
F25	PZ245563	*Trichoderma spirale* DAOM 183974 (NR_077177.1)	100	0.0	99.18	635
F35	PZ245567	*Aspergillus versicolor* ATCC 9577 (NR_131277.1)	99	0.0	99.82	591
F40	PZ245568	*Aspergillus pseudoglaucus* NRRL 40 (NR_135336.1)	98	0.0	100.00	575
F42	PZ260816	*Aspergillus niger* strain AN0512 (LC541742.1)	99	0.0	99.70	1669
F46	PZ260817	*Fusarium oxysporum* isolate FGZ12 (ON312900.1)	99	0.0	99.78	1352
F52	PZ245569	*Penicillium citrinum* NRRL 1841 (NR_121224.1)	96	0.0	99.07	574
F59	PZ245570	*Cladosporium colombiae* CBS 274.80B (NR_119729.1)	97	0.0	98.89	653
F61	PZ245571	*Cladosporium endophyticum* MFLUCC 17-0599 (NR_158360.1)	99	0.0	98.56	581
F63	PZ260818	*Alternaria* sp. isolate T1 (ON876488.1)	99	0.0	99.85	1659
F71	PZ260819	*Fusarium solani* isolate F4P2Rc (ON026100.1)	99	0.0	99.63	1364

**Table 3:** Inhibition rate of antagonistic endophytes to three kinds of the fungal pathogens of *P. cyrtonema* Hua.

Endophytes Strains	Inhibition Rate of Fungal Colony Growth (%)		
	F2	F3	F8
3-11	65.51 ± 3.67	78.04 ± 1.08	65.36 ± 5.40
3-29	66.11 ± 2.77	76.99 ± 3.18	67.71 ± 2.88
3-62	54.91 ± 5.13	78.48 ± 2.58	56.41 ± 2.21
3-65	66.35 ± 2.31	79.33 ± 2.42	61.66 ± 0.52
T26-2	60.18 ± 2.22	71.49 ± 1.72	67.43 ± 2.32
T45-2	63.50 ± 1.47	77.60 ± 3.88	58.35 ± 0.54

**Table 4:** Inhibition rate of fermentation supernatant of antagonistic endophytes to three kinds of the fungal pathogens of *P. cyrtonema* Hua.

Endophytic Strains	Inhibition Rate of Fungal Colony Growth (%)		
	F2	F3	F8
3-11	80.11 ± 0.00	90.00 ± 0.00	74.34 ± 0.76
3-29	78.99 ± 0.00	90.00 ± 0.00	77.96 ± 1.36
3-62	76.11 ± 1.56	87.42 ± 0.12	73.59 ± 0.27
3-65	77.03 ± 0.20	90.00 ± 0.00	79.03 ± 1.02
T26-2	80.20 ± 0.81	90.00 ± 0.00	79.52 ± 0.34
T45-2	76.66 ± 0.33	90.00 ± 0.00	76.86 ± 1.18

## Data Availability

The data presented in this study are available in this article and on request from the corresponding author.
